# Ki67 Immunohistochemical Expression Level ≥70%, Bulky Presentation ≥7.5 cm, Meningeal Lymphomatosis, and Interim PET ΔSUVmax After 4 Treatment Cycles <71% as Parts of a Practical Scoring System to Predict Progression-Free Survival and Overall Survival in Diffuse Large B-Cell Lymphoma

**DOI:** 10.3389/fnume.2022.829138

**Published:** 2022-04-07

**Authors:** Vincent Rebière, Meriem Maajem, Ronan Le Calloch, Leela Raj, Anne-Sophie Le Bris, Mohamed Malou, François Salmon, Isabelle Quintin-Roué, Adrian Tempescul, David Bourhis, Laura Samaison, Hussam Saad, Pierre-Yves Salaun, Christian Berthou, Jean-Christophe Ianotto, Ronan Abgral, Jean-Richard Eveillard

**Affiliations:** ^1^Department of Hematology, Brest University Hospital, Brest, France; ^2^Department of Nuclear Medicine, Brest University Hospital, Brest, France; ^3^Department of Internal Medicine, Blood and Infectious Diseases, Cornouaille Hospital Center, Quimper, France; ^4^Faculty of Health Science, McMaster University, Hamilton, ON, Canada; ^5^Department of Internal Medicine, Michel Mazéas Hospital Center, Douarnenez, France; ^6^Department of Hematology and Oncology, Morlaix Hospital Center, Morlaix, France; ^7^Department of Nuclear Medicine, Cornouaille Hospital Center, Quimper, France; ^8^Department of Anatomo-Pathology, Brest University Hospital, Brest, France; ^9^Department of Anatomo-Pathology, Cornouaille Hospital Center, Quimper, France

**Keywords:** DLBCL, survival, Ki67, bulky, meningeal lymphoma, PET-CT, scoring system

## Abstract

Currently, prognostic models in diffuse large B-cell lymphoma (DLBCL) fail to closely reflect patients' biological, clinical, and survival heterogeneity. We, therefore, assessed the impact of clinical, biological, immunohistochemical (IHC), baseline (0), and interim (after 2 and 4 treatment cycles) PET (PET0, PET2, and PET4) data not yet included in any scoring system on DLBCL outcome. The analysis was conducted on 89 previously untreated adult patients of the Finistere Observatory Cohort (O.Ly.Fin) with documented DLBCL, recruited between January 2010 and December 2017, with progression-free survival (PFS) and overall survival (OS) as primary and secondary endpoints, respectively. Seventy-eight patients were treated with rituximab, cyclophosphamide, hydroxyadriamycin, vincristine, and prednisone (R-CHOP), while 11 received R-dose-adjusted etoposide, prednisone, vincristine, cyclophosphamide, and hydroxyadriamycin (EPOCH). Patients were followed up until June 20, 2020. On multivariate analysis, Ki67 ≥ 70% on IHC (K), bulky presentation ≥7.5 cm (B), meningeal lymphomatosis (M), and PET0–PET4 ΔSUVmax <71% (P4) were identified as strong independent predictors of PFS, and all variables but bulky disease also strongly and independently predicted OS. Using these 4 parameters, we designed a scoring model named KBMP4 stratifying patients into low- (0 parameter), intermediate- (1 or 2), and high-risk (≥3) subgroups by the Kaplan–Meier analysis. At a median follow-up of 43 months, PFS and OS were both 100% in the low-risk subgroup, 71.4 and 90.5%, respectively, in the intermediate-risk subgroup, and 0 and 55.5%, respectively, in the high-risk subgroup. Use of the KBMP4 model in clinical practice may improve accuracy in prognostic prediction and treatment decisions in *de novo* DLBCL patients.

## Introduction

Diffuse large B-cell lymphoma (DLBCL) is the most common subtype of B-cell lymphoma in western countries, accounting for 30% of non-Hodgkin's lymphomas (NHL) in adults (1). The major prognostic scores for *de novo* DLBCL are the International Prognostic Index (IPI) and the age-adjusted (aa) IPI for patients under 60 years (2). IPI is based on clinical and biological factors including age >60 years, Ann Arbor stage III or IV, serum lactate dehydrogenase (LDH) above the upper normal limit, Eastern Cooperative Oncology Group (ECOG) Performance Status (PS) ≥2, and >1 extranodal site involvement. Each factor is attributed one point. Patients are stratified into low (0–1 point), low-intermediate (2 points), high-intermediate (3 points), and high-risk (≥4 points) categories accordingly with respective 3-year overall survival (OS) of 91%, 81%, 65%, and 60%.

High-grade B-cell lymphomas (HGBL) are a new category of aggressive B-cell lymphomas introduced by the WHO in 2016 (1). Specifically, HGBL may be subclassified as “double hit” (DH), involving chromosomal breakpoints on MYC and BCL2 or BCL6 genes on molecular analysis, or as “triple hit” (TH), implying concomitant chromosomal breakpoints on MYC, BCL2, and BCL6 genes. In patients presenting with overexpression of MYC and BCL2 proteins on immunohistochemical (IHC) analysis, some DLBCLs not otherwise specified (NOS) have been defined as dual-expressor (DE) lymphoma. In all cases, DH, TH, and DE statuses portend a poor prognosis (3–8).

Additional evidence predicts unfavorable outcomes and chemoresistance in DLBCL patients. This includes subclassification into the germinal center (GC) or non-GC DLBCL based on the cell of origin (COO) (9–11), high SUVmax (12), high total metabolic tumor volume (TMTV) (13), and high total lesion glycolysis (TLG) (14) on baseline PET imaging and failure to reach complete metabolic response as assessed by visual [International Harmonization Project (IHP) and Deauville 5-point scale (5-PS)] (15) or semiquantitative (ΔSUVmax) methods with interim PET evaluation after 2 and 4 treatment cycles (16). Regarding interim PET, although IHP, Deauville 5-PS, and ΔSUVmax have shown significant predictive value, ΔSUVmax was reported as an independent prognostic factor and is presumed to offer more advantages (17, 18).

Despite widely recognized pathobiological heterogeneity across cases, R-CHOP (rituximab, cyclophosphamide, hydroxyadriamycin, vincristine, and prednisone) regimen remains the primary approach in most DLBCL patients as uniform first-line immunochemotherapy (19). However, while 60% of patients treated with R-CHOP are considered cured without early or late relapse, 10% are primary refractory with a very poor prognosis, and 25% of those under 65 years are considered late responders and candidates for intensification followed by autologous stem cell transplantation (ASCT) (20). Important uncertainty remains regarding the 30–35% of patients who failed to respond to R-CHOP as the first line. Infusional R-dose adjusted (DA)-EPOCH (rituximab, etoposide, prednisone, vincristine, cyclophosphamide, and hydroxyadriamycin) regimen, once considered a better upfront option in patients with MYC-rearranged DLBCL (21), finally showed no superiority in survival to R-CHOP in this population in a recent large retrospective review (22). The optimal management of such patients remains debatable, and no reliable prognostic scoring systems based on the above-mentioned biomarkers currently exist to identify them early (23–25).

In the present study, we explored the prognostic value of several variables in DLBCL patients, including demographic, clinical, biological, and IHC data, as well as PET biomarkers at baseline and following 2 and 4 courses of immunochemotherapy. Based on our results, we designed a prognostic scoring model in DLBCL named KBMP4, which appears promising for earlier and more effective identification of patients susceptible to be unresponsive to R-CHOP.

## Methods

### Design

This is a retrospective multicenter observational study involving the West Brittany Inter-Hospital Federation (FIHBO) (Brest University Hospital, Quimper Hospital Center, and Douarnenez Hospital Center) and the Finistere Observatory Cohort (O.Ly.Fin).

This study was approved by our institution's ethics committee and conducted in accordance with the Declaration of Helsinki.

Eligible patients had documented *de novo* DLBCL (26) for which they had undergone PET at baseline and interim assessment after 2 and 4 cycles of chemotherapy.

**Figure 1:**
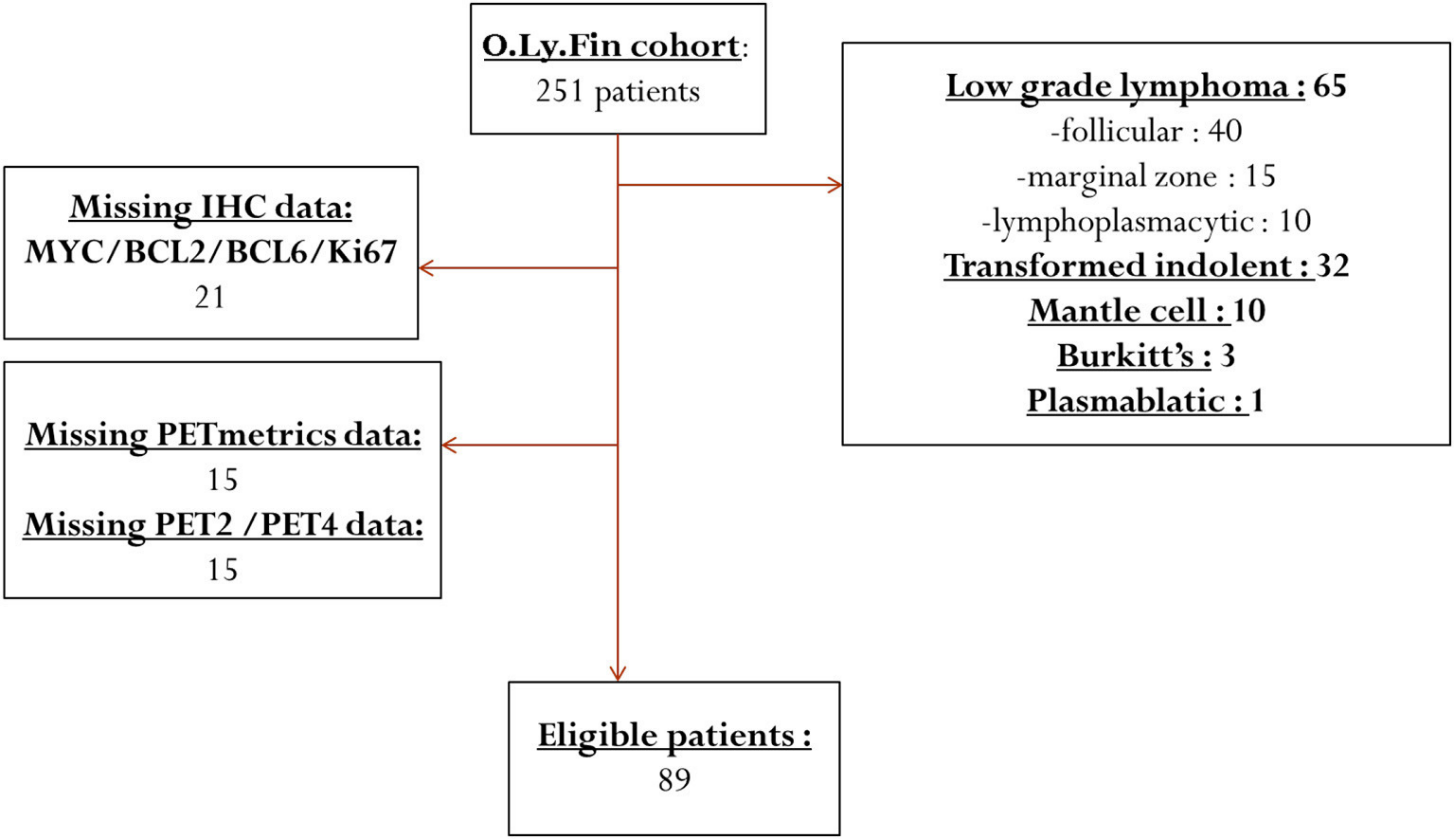
Flow chart of the exclusion process of initially selected patients.

Among 251 patients, identified consecutively from January 2010 to December 2017, 89 were eligible for inclusion in the analysis (Figure 1).

Optimal treatment for each patient was determined by a board of expert clinicians based on age, PS, IPI score, and evidence of poor prognostic features (MYC-positive IHC staining, bulky presentation defined as a tumoral lesion with diameter ≥7.5 cm, and extranodal involvement).

All responding patients received 6 R-CHOP-based immunochemotherapy cycles. Patients having tested positive for MYC received R-(DA)-EPOCH.

Demographic, clinical, biological, PET, and follow-up (FU) data were collected from patient files (see Supplementary Material for details).

Patients were followed up for at least 6 months, or until the occurrence of an event, to calculate progression-free survival (PFS) and OS. Clinical FU visits were scheduled and recorded according to European Society for Medical Oncology (ESMO) guidelines (27).

### Imaging Techniques and Analysis

CT was initially cranio-caudally performed with a whole-body protocol and injection of iodized contrast material (1.5 ml/kg) in the absence of contraindication. Whole-body PET/CT data were acquired in 3D mode and included both emission images (2 to 3 min per step) and transmission images required for attenuation correction (see Supplementary Material for details).

Quantitative SUV-based parameters were collected for each patient (see Online Supplementary Data for details).

PET/CT scanners in our institutions have EANM Research Ltd. (EARL) accreditation, in accordance with our policy regarding compliance with best clinical and technical guidelines, in daily practice as well as within the frame of international clinical trials.

### Statistical Methods

Continuous variables were compared using Student's t-test, ANOVA, or Mann–Whitney non-parametric test. Categorical variables were compared using the chi-squared test or Fisher's exact test, as appropriate.

PFS was measured from the date of diagnosis to the date of relapse, death, or last FU. OS was calculated from the date of diagnosis to the date of death or last FU. The ΔSUVmax PET0–PET2 <66% and ΔSUVmax PET0–PET4 <71% thresholds, as defined in a recently reported phase 3 study by LYSA, were used, but we did not apply the Deauville 5-PS as recommended by the 2011 Menton workshop, as <10% of patients met the criterion of PET0 SUVmax <10 (28).

Variables significant (*p* < 0.10) on univariate analysis were included in the multivariate analysis. PFS and OS were calculated for all patients and for patient subgroups, divided through receiver operating characteristic (ROC) analysis, using the Kaplan–Meier survival analysis, and compared between subgroups using a log-rank test.

A multivariate Cox regression analysis was performed to test the association between variables and survival rates. All tests were two-sided, and a *p* < 0.05 was considered statistically significant.

Based on the results, we stratified patients into three prognostic subgroups according to the number of risk factors: 0 (A), 1 or 2 (B), or ≥3 (C).

Statistical analyses were performed using Addinsoft 2020 XLSTAT 2020.

## Results

### Patient Characteristics

#### Demographic, Clinical, Biological, and Prognostic Characteristics

Table 1 summarizes patients' baseline demographic, clinical, and biological characteristics in addition to lymphoma IHC, bio-clinical, baseline and interim PET features, and IPI-based prognostic profiles at diagnosis.

**Table 1:** Patients baseline demographic, clinical, biological, PET and prognostic characteristics.

**Parameters**	**Patients with *de novo* DLBCL (*n* = 89)**
	**Value (%)**
Age	
Median (range), years	61.2 (18.3–79.2)
Gender	
Male	55 (61.8%)
Female	34 (38.2%)
Body Mass Index	
A: ≤ 20	10 (11.2%)
B: 21-25	27 (30.3%)
C: 26-30	34 (38.2%)
D: ≥ 31	18 (20.2%)
ECOG performans status	
0	40 (44.9%)
1	37 (41.6%)
2	11 (12.3%)
3	1 (1.1%)
B symptoms	36 (40.4%)
Ann Arbor stage ≥ 3	77 (86.5%)
Bulky presentation ≥ 7.5 cm	43 (48.3%)
International Prognostic Index	
0	1 (1.1%)
1	12 (13.5%)
2	23 (25.8%)
3	28 (31.5%)
>3	25 (28.1%)
Meningeal lymphomatosis	3 (3.4%)
Baseline blood biochemistry	
LDH > upper normal limit	58 (65.2%)
β-2-microglobuline ≥ 2.5 mg/L	68 (76.4%)
Immunohistochemistry	
GC^*^ status	45 (50.6%)
MYC+	18 (20.2%)
BCL2+	64 (71.9%)
BCL6+	64 (71.9%)
Ki67 ≥ 70%	66 (74.1%)
First line treatment	
R-CHOP	78 (87.6%)
R-DA-EPOCH	11 (12.4%)
Baseline PET SUVmax (X)	
X ≤ 15	20 (22.5%)
16 < X ≤ 25	21 (23.6%)
26 < X ≤ 40	31 (34.8%)
X ≥ 41	17 (19.1%)
Interim PET^*^ΔSUVmax	
PET0-PET2 <66%	74 (83.1%)
PET0-PET4 <71%	70 (78.6%)

*Data are shown as number of patients affected (% of total number of patients with documented data), unless otherwise specified (*

* *GC, germinal center; PET, positron-emission tomography; Δ, delta)*.

#### Immunohistochemical Characteristics

According to the Hans algorithm, 45 patients (51.7%) had GC-subtype DLBCL. Through IHC expression, MYC was positive in 18 (20.2%), BCL2 in 64 (71.9%), and BCL6 in 64 (71.9%). Median IHC positivity for Ki67 was 80%, ranging from 20 to 100%, and in 66 patients (74.1%), Ki67 positivity was ≥70%.

#### PET Characteristics

The mean baseline SUVmax was 30.85, ranging from 2 to 57. Mean PET0–PET2 ΔSUV was 74.7% (0–97.4%), and 74 patients (83.1%) were above the cutoff of <66%. Mean PET0–PET4 ΔSUV was 78.1% (0–98.1%), and 70 patients (78.6%) were above the cutoff of <71%. Of note, among 19 patients whose PET0–PET4 ΔSUVmax was <71%, histological assessment and PET4 proofreading were undertaken in 6 who were in fact and are still in complete response (CR) without additional treatment, while 13 others were not in CR and underwent salvage therapy. For the avoidance of doubt in these 19 patients, the response was also assessed according to Deauville 5-PS and was reported as follows: of the six responders, 5-PS was estimated to be 5 in 1, 4 in 1, 3 in 1, and 2 in 3; of the 13 nonresponders, it was evaluated to be 5 in 6, 4 in 2, 2 in 1, and 1 in 4.

### Treatment Outcome and Follow-Up

As detailed below, 84 (92%) patients responded to treatment, 76 patients were in CR following the first line, and 8 experienced CR after salvage treatment.

First-line treatment with R-CHOP in 78 patients (87.6%) and R-(DA)-EPOCH in 11 (12.4%) resulted in CR in 76 (85.4%), partial response (PR) in 6 (6.7%), and failure in 7 (7.9%). The six patients in PR proceeded to ASCT, after R-DHAP as salvage treatment in three and no salvage in three; after the procedure, two were in failure and four advanced to CR. Among the seven patients in failure, four advanced to CR: one after R-DHAP followed by ASCT as salvage strategy and three after rescue by rituximab, dexamethasone, high-dose aracytine, platinol (cisplatin) (R-DHAP), rituximab, etoposide, solumedrol, aracytine, platinol (cisplatin) (R-ESAP), and rituximab, dexamethasone, high-dose aracytine, oxaliplatin (R-DHAOX); the other three remained in failure after two or three further treatment lines. No ASCT was performed in patients over 68 years old.

Patients were followed up until June 20, 2020. Median FU was 43 months, ranging from 6.9 to 101.1 months. FU length was >36 months in 60 (67.4%), >48 months in 40 (45%), and >60 months in 26 (29.2%). No patients were lost to FU.

A total of 27 relapse events were observed. Overall median time to relapse was 8.2 months, ranging from 1.9 to 65.9. Early relapse, defined as disease recurrence within the year of diagnosis or 6 months after the end of treatment, was observed in 16 patients (59.3%), whereas late relapse, defined as disease recurrence later than 1 year after diagnosis, occurred in 11 (40.7%).

A total of 11 patients died during FU: nine from disease-related causes (five in primary failure, one in early relapse, and three in late relapse) and two from unrelated causes. Overall median time to death was 14.5 months, ranging from 6.9 to 69.8.

### Survival Analysis

#### Progression-Free Survival

On multivariate Cox model, bulky presentation (*p* = 0.030), meningeal lymphomatosis (*p* = 0.020), Ki67 ≥ 70% (*p* = 0.007), and PET0–PET4 ΔSUV <71% (*p* = 0.005) demonstrated statistical significance for PFS (Table 2). Despite a *p*-value of 0.083 in univariate analysis, IPI > 2 failed to show statistical significance while aaIPI > 1, MYC status, and GC/non-GC profile were not eligible for multivariate analysis.

**Table 2:** Multivariate Cox model analysis for PFS.

**Variables**	**Univariate**	**Multivariate**	
	***p***	**HR (95% CI)**	***p***
Gender (male)	0.085	-	-
Bulky presentation ≥7.5 cm	0.007	2.594 (1.097–6133)	0.030
Meningeal lymphomatosis	0.009	6.827 (1353–34.447)	0.020
IPI ≥ 3	0.083	-	-
Ki67 ≥ 70%	0.056	4.102 (1.470–11.444)	0.007
PET0-PET2 ΔSUV <66%	0.018	-	-
PET0-PET4 ΔSUV <71%	0.001	5.677 (1.684–19.144)	0.005

Eligible parameters identified by univariate analysis with p < 0.10 (* IPI, International Prognostic Index; PET, positron-emission tomography; Δ, delta).

**Figure 2:**
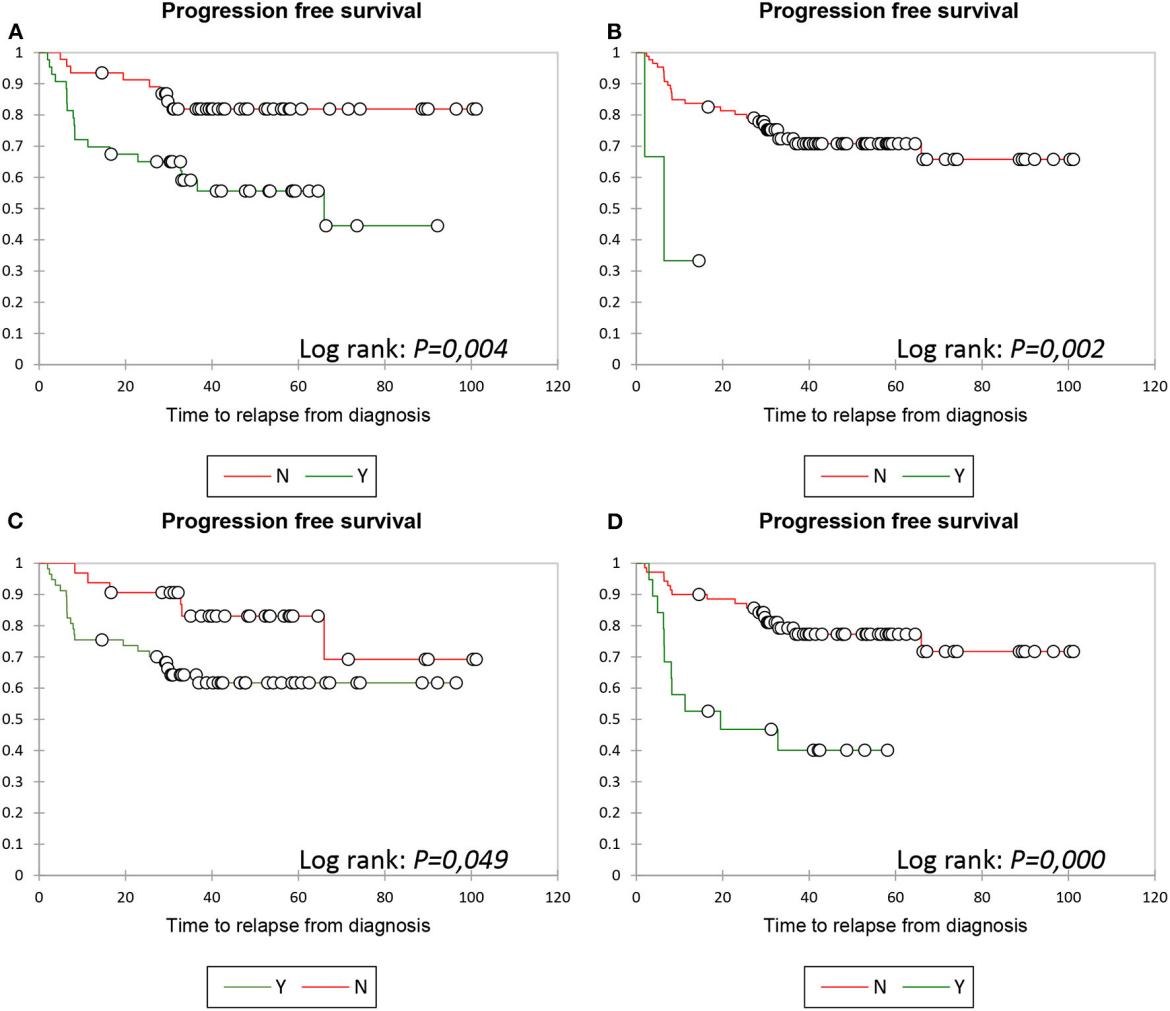
PFS: Kaplan Meier analysis. PFS according to bulky presentation ≥7.5 cm **(A)**, meningeal lymphomatosis **(B)**, Ki67 ≥70% **(C)** and PETO-PET4 ΔSUV <66% **(D)**, respectively.

Kaplan–Meier analysis showed a statistically significant difference in PFS between patients with and without bulky presentation ≥7.5 cm (*p* = 0.004) (Figure 2A), with and without meningeal lymphomatosis (*p* = 0.002) (Figure 2B), with and without Ki67 ≥ 70% (*p* = 0.049) (Figure 2C), and with and without PET0–PET4 ΔSUV <71% (*p* = 0.000) (Figure 2D). PFS at 24 months of FU (PFS24) was 65 vs. 89% in patients with or without bulky presentation, 70 vs. 87.5% in patients with or without Ki67 ≥ 70%, 0 vs 79% in patients with or without meningeal lymphomatosis, and 47.3 vs. 85.7% in patients with or without PET0–PET4 ΔSUV <71%.

A prognostic scoring model, based on the identified predictors, was designed and named KBMP4 (Ki67, Bulky, Meningeal disease, PET4). No patients had all 4 parameters. With the use of Kaplan–Meier analysis (**Figure 4 left**), three distinct subgroups were identified based on the number of risk factors: in subgroup A (low-risk with 0 parameters), no relapse was observed in 17 patients (0%); in subgroup B (intermediate risk with 1 or 2 parameters), relapse occurred in 18/63 patients (28.6%), with a median time of 21.1 months, ranging from 2.4 to 65.9; in subgroup C (high risk with ≥3 factors), relapse was observed in 9/9 patients (100%), with a median time of 6.3 months, ranging from 1.9 to 8.2. A significant difference was noticed when subgroup A was compared to subgroup B (*p* = 0.014), subgroup A to group C (*p* < 0.0001), and subgroup B to subgroup C (*p* < 0.0001). PFS24 was 100% in subgroup A, 81% in subgroup B, and 0% in subgroup C.

#### Overall Survival

Multivariate Cox model identified age at diagnosis (*p* = 0.005), meningeal lymphomatosis (*p* = 0.000), Ki67 ≥ 70% (*p* = 0.007), PET0–PET2 ΔSUV <66% (*p* = 0.045), and PET0–PET4 ΔSUV <71% (*p* = 0.008) as the statistically significant parameters negatively influencing OS as shown in Table 3. Despite a *p*-value of 0.0041 and 0.121, respectively, in univariate analysis, MYC status and IPI > 2 failed to show statistical significance, while aaIPI > 1 and GC/non-GC profile were not eligible for multivariate analysis.

**Table 3:** Multivariate Cox model analysis for OS.

**Variables**	**Univariate**	**Multivariate**	
	***p***	**HR (95% CI)**	**p**
Age	0.015	1.184 (1.054–1.330)	0.005
ECOG PS^*^ ≥ 2	0.031	-	-
Meningeal lymphomatosis	0.002	527.375 (18.289–15207.407)	0.000
MYC IHC^*^ overexpression	0.041	-	-
Ki67 ≥ 70%	0.083	130.129 (3.903–4339.114)	0.007
PET^*^0-PET2 Δ^*^SUV <66%	0.011	6.530 (1.042–40.912)	0.045
PET0-PET4 ΔSUV <71%	0.006	18.520 (2.174–157.789)	0.008

*Eligible parameters identified by univariate analysis with p < 0.10 (*

* *PS, positron-emission tomography; Δ, delta)*.

**Figure 3:**
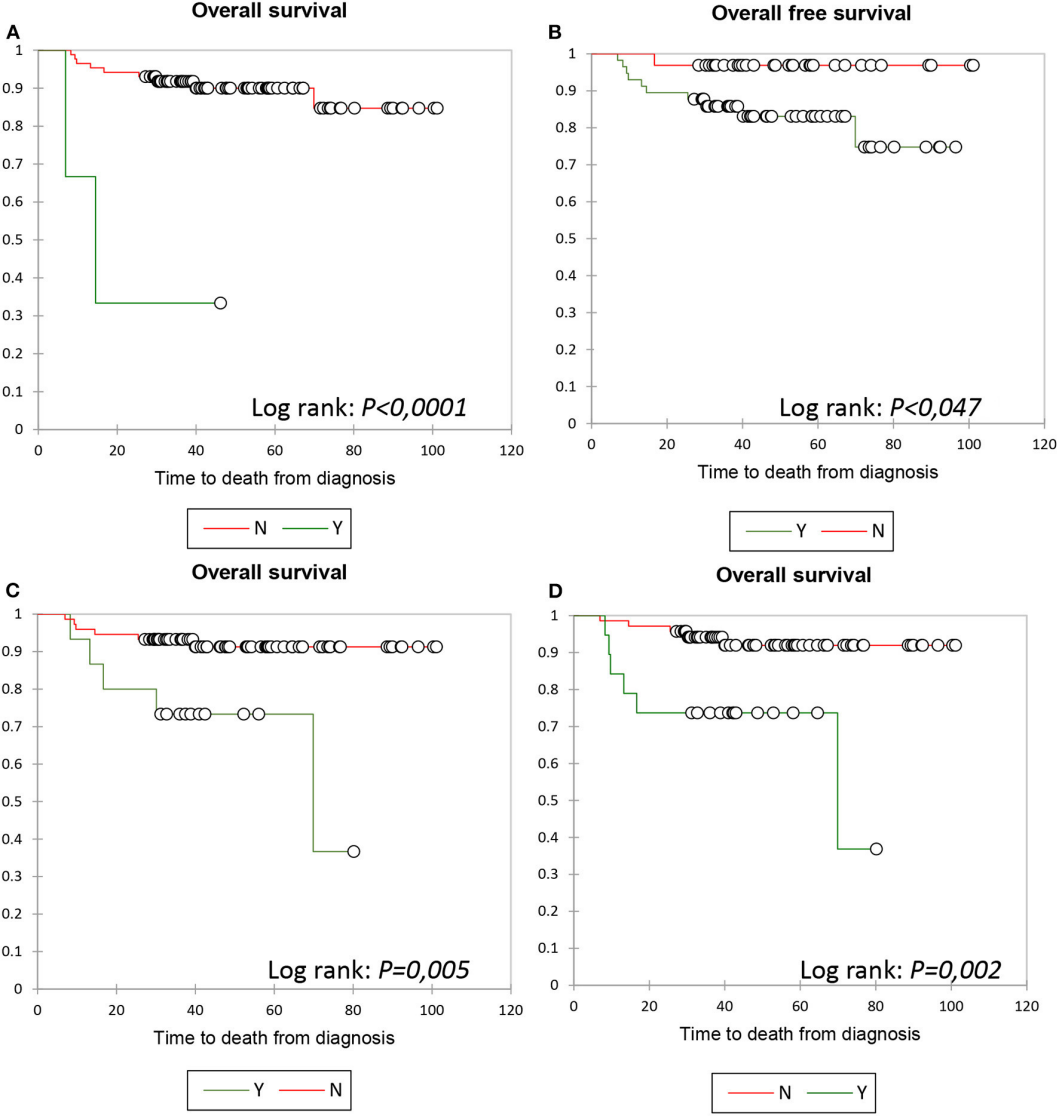
PFS: Kaplan Meier analysis according to the KBMP4 scoring model. OS according to meningeal lymphomatosis **(A)**, Ki67 ≥70% **(B)**, PETO-PET2 ΔSUV <66% **(C)** and PETO-PET4 ΔSUV <71% **(D)**, respectively.

The Kaplan–Meier analysis showed a statistical significance in OS regarding age (*p* = 0.017) and a statistically significant difference between patients with or without meningeal lymphomatosis (*p* < 0.0001), with or without Ki67 ≥ 70% (*p* = 0.047), with or without PET0–PET2 (P2) ΔSUV <66% (*p* = 0.005), and with or without PET0–PET4 ΔSUV <71% (*p* = 0.002) (Figure 3; data regarding age not shown).

**Figure 4:**
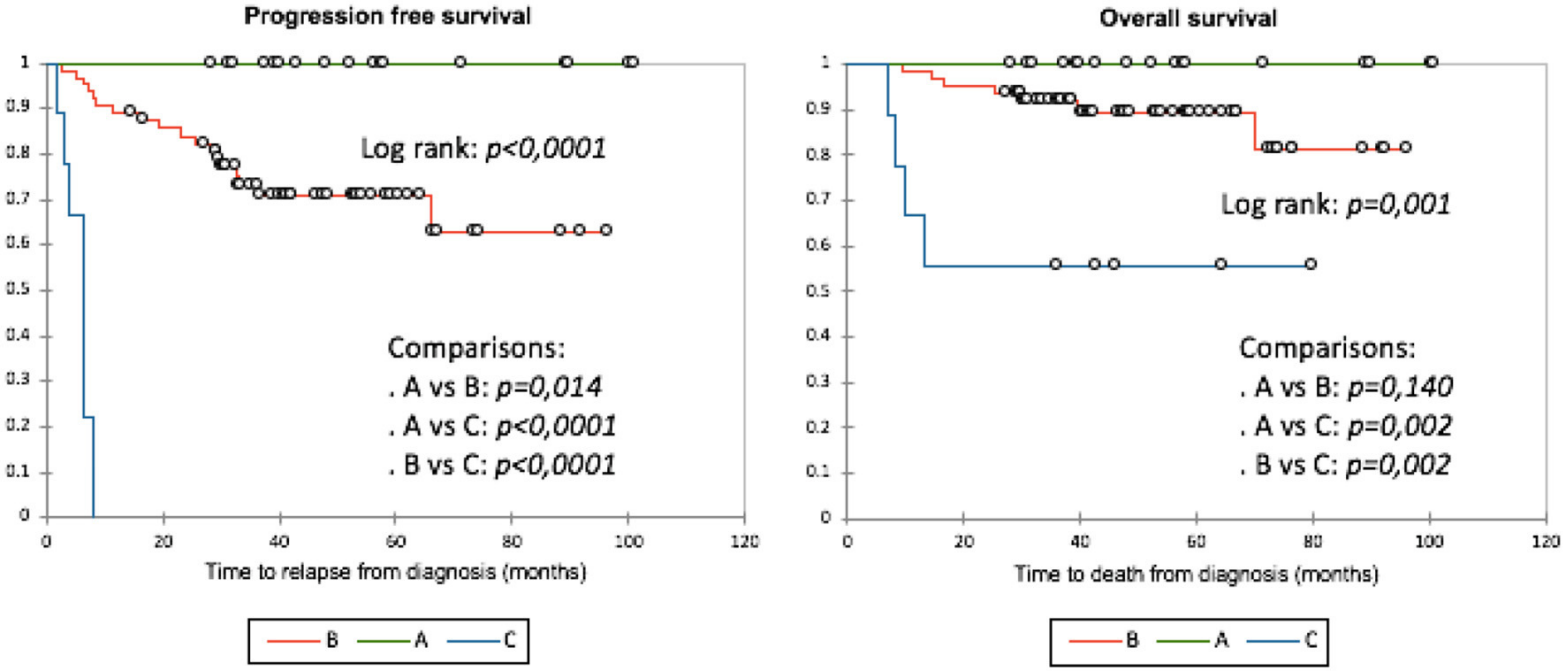
PFS and OS according to KBMP4 scoring models: Kaplan Maier analysis. KBMP4 scoring model identified three distint prognostic low-(A: *O* parameter), intermediate- (B: 1 or 2 parameters) and high risk (C: ≥3 parameters) subgroup regarding PFS (**left**) as well as OS (**right**).

As KBMP4 parameters, except for bulky presentation ≥7.5 cm, were found statistically significant in multivariate analysis, the model was tested and showed prognostic significance for OS: in subgroup A, no patient out of 17 died (0%); in subgroup B, 6/63 patients (9.5%) died, with a median time to death of 21.9 months, ranging from 9.3 to 69.8; and in subgroup C, 4/9 patients (44.4%) died, with a median time to death of 9 months, ranging from 6.9 to 13.2. No statistical significance was noticed when subgroup A was compared to subgroup B (*p* = 0.140), while a significant difference was observed when comparing subgroup A to subgroup C (*p* = 0.002) and subgroup B to subgroup C (*p* = 0.002) (Figure 4 **right**).

## Discussion

DLBCL is a set of highly aggressive NHLs with variable and heterogeneous prognoses. IPI and aaIPI are currently the most widely accepted prognostic indices to stratify DLBCL patients (2). Nevertheless, several drawbacks exist in the use of IPI and aaIPI. Firstly, these indices were designed prior to the introduction of rituximab. Additionally, their ability to adequately and finely identify patients at risk of treatment failure in the rituximab era, in terms of both PFS and OS, is still being debated (29, 30). Currently, 30%−35% of patients who are treated by R-CHOP in the first line will experience a poor outcome with early or late relapse or primary refractoriness. This evidence, therefore, suggests the need for a more sensitive prognostic score able to identify patients at risk for adverse outcomes and to adjust the treatment approach accordingly (23, 31).

In our present analysis, we identified four independent and routinely collected biomarkers, namely, Ki67 ≥ 70%, bulky presentation ≥7.5 cm, meningeal lymphomatosis, and ΔSUVmax PET0–PET4 <71%, which were highly predictive of PFS, the main predictor of treatment success.

The KBMP4 scoring model robustly discriminates patients' PFS into low- (0 factors), intermediate- (1 or 2), and high-risk (≥3) subgroups. In the low-risk subgroup, 100% of patients remained relapse-free, whereas in the high-risk subgroup, 100% of patients relapsed, within a median time of 6.3 months, ranging from 1.9 to 8.2. Of note, although all but 1, namely, bulky presentation, of the four key parameters of the model proved successful in predicting OS, likely due in part to the low death rate, the model clearly showed significant differences in OS across the three subgroups, with 0% of death in the low-risk subgroup and 44.4% of high-risk patients dying in a median time of 9 months, ranging from 6.9 to 13.2. Contrastingly, when applied to our cohort of patients, IPI > 2 and aaIPI > 1 failed to show statistical significance for both PFS and OS.

With respect to biological features, IHC-defined GC/non-GC subclassification based on the Hans algorithm and DE profile along with molecularly defined DH/TH status have been suggested to have prognostic significance (3, 8, 10). DH/TH status is not routinely assessed in our institutions and was, therefore, not tested for predictive ability. MYC status, limited to DE profile in our cohort, did not show statistical significance for OS. Nonetheless, it is worth mentioning that, among 11 deceased patients, there were 5 MYC+ in whom KBMP4 was low in 1, intermediate in 3, and high in 1; there were 6 MYC– in whom KBMP4 was intermediate in 3 and high in 3. Non-GC lymphomas, with a higher trend toward extranodal involvement and meningeal lymphomatosis, are reportedly more aggressive than GC lymphomas (32). However, 10% to 20% of cases are “unclassifiable” according to the Hans algorithm, whose predictive value for survival is currently being debated (33). In our study, these parameters had no significant predictive value regarding PFS or OS. In contrast, consistent with previous studies (34), our results revealed that Ki67 IHC expression ≥70% was associated with an unfavorable impact on PFS and OS.

The adverse prognostic impact of bulky presentation in DLBCL is widely reported, notably in case of failure to reach CR on interim PET, which can be reversed by involved-field radiotherapy as demonstrated by the UNFOLDER trial (35). But the defining tumor-size range varies from 5–6 to 10 cm among studies (31). According to the National Comprehensive Cancer Network, bulky disease should be defined as a tumor ≥7.5 cm in diameter (36). In our study, the cutoff was also set at 7.5 cm, and this parameter was found statistically significant for PFS, in both univariate and multivariate Cox model analyses, but not for OS.

Central nervous system (CNS) involvement, usually an exclusion criterion in most clinical trials, is responsible for high morbidity and shortened OS (37). It is, therefore, a strong indicator of early relapse and fatal outcomes. In this respect, our results confirm that meningeal lymphomatosis is associated with an unfavorable outcome and bring support to the standard approaches for CNS prophylactic or curative strategies in every newly diagnosed DLBCL (38).

Imaging through PET also holds a valuable place in discriminating low- and high-risk DLBCL patients and, most importantly, in the interim assessment of chemosensitivity. TMTV and TLG are not routinely assessed in our institutions. Quantitative measurement of ΔSUVmax between baseline PET0 and interim PET2 and PET4 is a novel approach for aggressive lymphoma, not universally accepted (39), but whose results are used to guide optimal treatment strategy (40). The optimal PET0–PET2 ΔSUVmax cutoff used to identify early and late responders to the R-CHOP regimen has been set at <66%, while the optimal PET0–PET4 ΔSUVmax cutoff used to identify nonresponders who will require salvage treatment has been set at <71% (16). Patients reaching complete metabolic response have a 2-year PFS > 70%. In accordance with published data, we observed a strong predictive value of PET0–PET4 ΔSUVmax for both PFS and OS, although we observed a false positivity rate of 31.5% in patients who could have been subject to unnecessary treatment intensification without histological assessment or PET4 proofreading. In contrast to reports in the medical literature, PET0–PET2 ΔSUVmax predictive value was limited to univariate analysis for PFS (41, 42), while in accordance with other studies, it was strongly demonstrated in multivariate and Kaplan–Meier analyses for OS (43). These results suggest the need for further investigation of PET0–PET2 ΔSUVmax predictive value.

The originality of the KBMP4 prediction model resides in its convenience and reproducibility, as it is based on robust prognostic factors, which are well described in the medical literature. Indeed, each parameter of the score is systematically collected at baseline, and PET is the major imaging technique in staging and response assessment, notably for all non-elderly patients. This score demonstrated a good ability to discriminate between low-, intermediate-, and high-risk patients, allowing for accurate predictions of PFS and OS.

Our results have potentially important implications for the management of newly diagnosed DLBCL patients. Firstly, our scoring scheme appears to offer improved DLBCL patient prognostic classification, allowing for enhanced foresight for physicians, patients, and their families and caregivers (44). Furthermore, early stratification according to prognosis and predicted response to treatment may offer ground to enroll patients in clinical trials exploring alternative regimens (45–47), especially those targeting new druggable molecules, with the hope to find out innovative treatments, including cellular therapy (48), better suited to the patient's profile than R-CHOP. Specifically, our findings may play a role in the context of the expected next generation of clinical trials based on the LYSA RT3 (Real Time Molecular Characterization of DLBCL, NCT03104478) study in the area of personalized treatment for cancer.

The main weaknesses of our study rest in the limited number of centers having been involved, resulting in small sample size, as well as the retrospective nature of our work. Furthermore, although MYC status was not found to be statistically significant regarding OS, it would be interesting to know if there were differences between subgroups of MYC+ and MYC– subjects, which was challenging due to the small number of MYC+ patients in our cohort. External validation of our prognostic model should be conducted to assess its reliability in a larger number of patients, including subjects meeting all the four key KBMP4 parameters, which may allow for further discriminating the intermediate-risk patients into low- and high-intermediate additional subgroups reflecting their prognostic profiles more accurately.

## Conclusion

In summary, KBMP4 is a promising prognostic scoring model that has demonstrated the ability to discriminate between low-, intermediate-, and high-risk DLBCL patients in our cohort, with respect to both PFS and OS. Ki67 ≥70%, bulky tumor ≥7.5 cm, meningeal involvement, and PET0–PET4 ΔSUVmax <71% were identified as independent predictors of relapse and, except for bulky tumor, of OS in patients who were newly diagnosed with DLBCL. The use of this score in clinical practice may allow for improved treatment guidance and tailored treatment for each patient.

## Data Availability Statement

The original contributions presented in the study are included in the article/Supplementary Material, further inquiries can be directed to the corresponding author.

## Ethics Statement

The studies involving human participants were reviewed and approved by Comité d'Ethique du CHU de Brest. The patients/participants provided their written informed consent to participate in this study.

## Author Contributions

VR collected data and references and wrote the manuscript. MMaa, P-YS, RA, FS, and DB reviewed PET-CT data. LR proofread the manuscript. IQ-R and LS reviewed histological data. RL, A-SL, and MMal enrolled patients and reviewed the manuscript. AT, HS, CB, and J-CI reviewed the manuscript. J-RE designed and directed the study, collected data, and wrote the manuscript. All authors contributed to the article and approved the submitted version.

## Conflict of Interest

The authors declare that the research was conducted in the absence of any commercial or financial relationships that could be construed as a potential conflict of interest.

## Publisher's Note

All claims expressed in this article are solely those of the authors and do not necessarily represent those of their affiliated organizations, or those of the publisher, the editors and the reviewers. Any product that may be evaluated in this article, or claim that may be made by its manufacturer, is not guaranteed or endorsed by the publisher.
