# Supplementary material for: Ki67 Immunohistochemical Expression Level ≥70%, Bulky Presentation ≥7.5 cm, Meningeal Lymphomatosis, and Interim PET ΔSUVmax After 4 Treatment Cycles <71% as Parts of a Practical Scoring System to Predict Progression-Free Survival and Overall Survival in Diffuse Large B-Cell Lymphoma
Source: Front Nucl Med. 2022 Apr 7;2:829138. doi: 10.3389/fnume.2022.829138 (PMC11440974; doi:10.3389/fnume.2022.829138)
Supplement: Supplementary file 1 [file Data_Sheet_1.PDF]

Supplementary Data:

Clinical and biological data and immunohistochemical analyses

Recorded clinical data included age, gender, body mass index (BMI), ECOG PS, B symptoms, Ann Arbor stage, bulky presentation ≥7,5 cm, IPI score, meningeal lymphomatosis, LDH and β-2-microglobuline levels and treatment regimens.

Recorded parameters for IHC analysis included distinction between B- or T-cell lineage, observation of possible variant forms, evaluation of proliferative index based on Ki67 IHC staining and assessment of EBV status on paraffin-embedded lymph node or other tissue biopsies. GC and non-GC subclassification was done according to Hans algorithm using CD10, BCL6 and MUM1/IRF4 expression[9]. DE status characterizing some DLBCLs NOS was routinely assessed using MYC and BCL2 immunostaining. Final histopathologic results were reported in accordance with the WHO 2016 classification[1].

Imaging Technique

Examinations were performed on several successive PET/CT hybrid machines within different centers: Gemini GXLi (Philips© Healthcare, Netherlands) and Biograph-mCT (Siemens©, Erlangen Germany) in Brest University Hospital; a Biograph system in Quimper General Hospital as well.

Standard patient preparation included a minimum 4-hour fast with serum blood glucose level <7 mmol/L before FDG administration. After an intravenous injection of 3-5 MBq/kg of 18-FDG (IBA Molecular Imaging©, Saclay, France), patients remained on bedrest for 1 hour. CT was initially performed in the cranio-caudally with a whole-body protocol and injection of iodized contrast material (1.5 mL/kg) in the absence of contraindication. Whole-body PET/CT data were acquired in 3D mode and included both emission images (2 to 3 minutes per step), and transmission images required for attenuation correction. The transmission images were obtained from the X-ray scan data. The emission images were corrected for background noise, random events and reconstructed with and without attenuation correction using the iterative LOR (=Line Of Response) RAMLA (Row-Action Maximum Likelihood Algorithm) method for Gemini system and the iterative Ordered Subset Expectation Maximization (OSEM) method for the Biograph systems. The PET images were smoothed with a Gaussian filter (width at half height = 2 mm). The 6-bar Gemini and 40-bar Biograph scanner had respectively 600 mm and 700mm transverse fields of view. The CT parameters were standard for PET/CT studies and permit differentiation between tissues with good spatial resolution while ensuring that the patient does not receive a high dose radiation.

Image analysis

Quantitative SUV-based parameters were collected for each patient on a Syngo.via® workstation (Siemens Healthineers©, Erlangen, Germany).

FDG uptake was obtained using SUVs, calculated according to the following formula: SUV = tissue radioactivity concentration [kBq/mL]/[injected dose (kBq)/patient weight (g)]. Spherical volumes of interest (VOI) were drawn over the most hypermetabolic lesion on PET0, PET2 and PET4, even if unmatched.

The maximal SUV (SUVmax), corresponding to the highest possible voxel uptake value in the VOI, were recorded.

The delta ΔSUVmax (ΔSUVmax) was calculated from the results of interim PET at 2 and 4 cycles according to the following equation:

ΔSUVmax (PET0-PET2) = [SUVmax (PET 2)-SUVmax (PET0)] / SUVmax (PET0).

ΔSUVmax (PET0-PET4) = [SUVmax (PET 4)-SUVmax (PET0)] / SUVmax (PET0).
